# Fatty acid profiles unveiled: gene expression in Yanbian yellow cattle adipose tissues offers new insights into lipid metabolism

**DOI:** 10.5194/aab-67-469-2024

**Published:** 2024-10-07

**Authors:** Azher Nawaz, Junzheng Zhang, Ying Meng, Lefan Sun, Haiyang Zhou, Chunyin Geng, Haixing Liu, Yinghai Jin, Shuang Ji

**Affiliations:** 1 Department of Animal Science, College of Agriculture, Yanbian University, Yanji 133000,Jilin Province, PR China

## Abstract

*Objectives.* The objectives of this study were twofold:

1. to analyze the composition and content of fatty acids in various adipose tissues (including kidney, abdominal, subcutaneous, and omental) of Yanbian yellow cattle and to observe the morphology of adipocytes within these tissues and
2. to assess the level of expression of specific genes – kinase insert domain receptor (*KDR*), apolipoprotein L domain containing 1 (*APOLD1*), stearoyl-CoA desaturase 1 (*SCD1*), secreted frizzled-related protein 4 (*SFRP4*), fatty-acid-binding protein 5 (*FABP5*), and sterol carrier protein-2 (*SCP2*) – in different adipose tissues (kidney, abdominal, posterior belly, ribeye, prothorax, striploin, upper brain, and neck) of Yanbian yellow cattle.

*Method.* Castrated Yanbian yellow cattle, 24 months old, with identical genetic backgrounds and raised under the same breeding management conditions, were selected. The fatty acid composition and content were assessed using gas chromatography, while the size and diameter of adipocytes were analyzed via paraffin sectioning. The level of expression was determined using quantitative reverse transcription polymerase chain reaction (qRT-PCR). *Results.* In total, 16 distinct fatty acids were identified in abdominal adipose tissue. Additionally, henicosanoic acid (C21:0) and behenic acid (C22:0) were detected exclusively in subcutaneous adipose tissue. Caprylic acid (C8:0) was found in both kidney and omental adipose tissues. The size of individual adipocytes in kidney adipose tissue was notably larger compared to the adipocytes in the other three regions ( p<0.05 ). Regarding gene expression, *APOLD1* exhibits its highest expression in striploin adipose tissues ( p<0.05 ), while *SCD1* shows its peak expression in prothorax adipose tissues ( p<0.05 ). Moreover, both *FABP5* and *SCP2* demonstrate their highest level of expression in prothorax adipose tissue ( p<0.05 ). Furthermore, the level of expression of *KDR* and *SFRP4* across these seven adipose tissue regions exhibits significant differences ( p<0.05 ). *Conclusion.* In conclusion, Yanbian yellow cattle exhibit variations in both the composition and content of fatty acids across different adipose tissue depots, including the kidney, abdominal, subcutaneous, and omental regions. Moreover, adipocytes display distinct morphological differences across these tissue types. Furthermore, the level of expression of *KDR*, *APOLD1*, *SCD1*, *SFRP4*, *FABP5*, and *SCP2* varies significantly among adipose tissues located in the kidney, abdominal, posterior belly, ribeye, prothorax, striploin, upper brain, and neck regions.

## 1 Introduction

Beef, among various meat products, is known for its high nutritional value (Geiker et al., 2021). The quality and flavor of beef are largely determined by marbling, with a positive correlation between intramuscular fat content and the marbling pattern. Higher intramuscular fat typically leads to a greater amount of marbling (Nguyen et al., 2021; Liu et al., 2021). In ruminants, adipose tissue serves as the primary site for de novo synthesis of fatty acids and triglycerides, and it is a major site for fat accumulation (Tan and Jiang, 2024; Urrutia et al., 2020). Factors influencing muscle fatty acids in ruminants are diverse and require careful regulation during production. Optimal meat quality is often associated with moderate intramuscular fat content, which is primarily stored in subcutaneous fat tissue (Pickworth et al., 2011). This study uses Yanbian yellow cattle to investigate adipocyte development, fatty acid composition, fat tissue content, and lipid metabolism gene expression in various adipose tissues. Yanbian yellow cattle are amongst the five most outstanding cattle breeds in China, well known for their strong body, exceptional cold resistance, ability to survive on rough feed, strong disease resistance, and tender flavorful meat. Keeping in mind the aforesaid qualities of Yanbian yellow cattle, we conducted an experiment on adipose tissue and fatty acid composition of Yanbian yellow cattle to further understand their unique features.

Fatty acids are vital for normal physiological functions and are classified into saturated fatty acids (SFAs) and unsaturated fatty acids. Unsaturated fatty acids are further categorized into monounsaturated fatty acids (MUFAs) and polyunsaturated fatty acids (PUFAs). Myristic acid (C14:0), palmitic acid (C16:0), and stearic acid (C18:0) are the common saturated fatty acids in beef fat (Uemoto et al., 2011). Polyunsaturated fatty acids (PUFAs) in beef, such as linolenic acid (C18:3n-3) and linseed oil acid (C18:2n-6), and MUFAs like oleic acid (C18:1n-9) are recognized for their cardiovascular benefits. Judicious consumption of MUFAs can decrease serum cholesterol and increase HDL (high-density lipoprotein) levels (Gilmore et al., 2013). The ratio of polyunsaturated fatty acid to saturated fatty acid (PUFA / SFA) is a critical indicator of the diet's impact on health, ratios of PUFA to SFA below 0.4 potentially increase circulatory lipid levels and the risk of cardiac diseases (Makarewicz-Wujec et al., 2018). Excessive intake of saturated fatty acids (SFAs) may weaken insulin sensitivity, a main factor in the metabolic syndrome and diabetes (Santaren et al., 2017). Compared to other ruminants, cattle adipose tissues have a firmer texture due to high concentration of saturated fatty acid. Conversely, in fattier cattle, the fat tends to be softer and more oily, which can be attributed to elevated ratios of 18:1 to 18:0 and 18:1 between fatty acids (Zechner et al., 2012).

Adipose tissue is a significant endocrine organ and a primary energy reservoir. The characteristics and secretory behaviors of fat vary among different fat deposits, particularly in visceral adipose tissue. Visceral fat cells and adipocytes differ in pathophysiological, regulatory, and functional aspects and should be analyzed separately (Titov, 2015). Low adiponectin levels can lead to autophagy in adipose tissue and abnormal adipocyte secretion, impacting insulin resistance and beta-cell dysfunction, making adiponectin a potential marker for various metabolic syndromes (Slutsky et al., 2016; Moon et al., 2019; Balsan et al., 2015). However, high adiponectin levels have been linked to increased cardiovascular mortality (Ortega Moreno et al., 2016).

Fat metabolism encompasses both synthesis and breakdown of fat, maintaining a dynamic equilibrium. In animals, triglycerides are primarily synthesized in the liver and adipose tissue, with fat deposition serving as a key energy storage mechanism (Takahashi et al., 2016). Fat deposition rates vary across different body parts and over time, leading to changes in body fat distribution with age (Hausman et al., 2018). Fat synthesis is controlled through two main mechanisms: direct regulation by enzyme activity and indirect regulation by metabolites and hormones. On the other hand, lipid breakdown is a physiological process that entails the hydrolysis of glycerol and free fatty acids to provide energy.

Gene expression in adipogenesis and adipocytes within adipose tissue are regulated by numerous transcription factors, influencing bovine adipocyte lipid metabolism. Genetic correlations suggest that certain fatty acids in beef tissue, carcass merit, and meat tenderness traits maybe influenced by a subset of common genes, including fatty acid synthesis (*FASN*), stearoyl-CoA desaturase (*SCD*), and fatty-acid-binding protein 4 (*FABP4*) in beef cattle (Ekine-Dzivenu et al., 2017). Notwithstanding their complexity, the roles played by various genes in lipolysis and lipogenesis are becoming clearer. For example, hormone-sensitive lipase (HSL) is a vital intracellular lipase found in adipose tissue that plays a critical role in the breakdown of lipid by hydrolyzing TAGs (triacylglycerols or triglycerides), DAGs (diacylglycerols), MAGs (monoacylglycerols), and CEs (cholesteryl esters) (Ochoa et al., 2004). In ruminants, acetyl-CoA carboxylase (ACC) has two isomers: ACC- α (*ACACA*), a rate-limiting enzyme in long-chain fatty acid synthesis, and ACC- β (*ACACB*), which controls fatty acid oxidation in mitochondria (Najafpanah et al., 2014). *ACACA* is excessively induced in adipose tissue production and nutritional regulation, with its mRNA expression correlating with total fat content in muscle, highlighting its significance in intramuscular fat deposition (Ehrlund et al., 2013; Mahdi et al., 2012). The *SREBP1* gene encodes a transcription factor vital for adipocyte differentiation and fatty acid synthesis (Laudes, 2011).

Lipogenesis contributes to understanding lipid metabolism in cattle which is vital for livestock industry as it can provide an insight into the fat composition of cattle which can influence meat quality and nutritional features (Schumacher et al., 2022). Moreover, investigation of lipid-metabolism-associated genes also helps in selective breeding programs (Abdollahi-Arpanahi et al., 2019). Certain genes are associated with a metabolic efficiency or desirable fatty acid profile; this information can be used in breeding strategies to improve meat quality and production efficiency (Zeng et al., 2023). Getting information about the fatty acid composition of Yanbian yellow cattle has potential implication for the nutritional quality of beef derived from these animals (X. Z. Li et al., 2018). Lipid profiling sheds light on the health and well-being of Yanbian yellow cattle because lipid metabolism is linked to the overall health, and understanding gene expression patterns will provide information about the metabolic health of cattle (Cho et al., 2023). Lipid profiling has an impact on the animal husbandry practices, optimization of feeding strategies, and enhancement of overall production efficiency (Song et al., 2022).

To elucidate the characteristics of Yanbian yellow cattle, we designed an experiment to investigate the fatty acid composition and content as well as the morphology of adipocytes in various adipose tissue depots, including kidney, abdominal, subcutaneous, and omental fat. Additionally, we quantified the level of expression of key genes involved in lipid metabolism, specifically *KDR*, *APOLD1*, *SCD1*, *SFRP4*, *FABP5*, and *SCP2*, in a range of adipose tissues, including kidney, abdominal, posterior belly, ribeye, prothorax, striploin, upper brain, and neck tissue. This comprehensive approach intended to deliver a detailed understanding of the lipid metabolism and adipose tissue biology in Yanbian yellow cattle.

## 2 Material and methods

### 2.1 Animal tissues

Yanbian yellow cattle used in this study were sourced from the national Yanbian yellow cattle core breeding farm, and selection was based on specific criteria. The selection criteria included

- age (the cattle were raised until they reached 24 months of age) and
- weight (the cattle had to weigh over 450 kg at the time of slaughter).

Samples were collected from ten 24-month-old castrated Yanbian yellow cattle at BenFu cattle farm, Longjing, ensuring similar feeding and genetic backgrounds. The animals were slaughtered, and the tissues were cut into small pieces, snap-frozen in liquid nitrogen, and stored at -80 °C for further analysis. All experiments were approved by the Yanbian University Institutional Animal Care and Use Committee (IACUC), with approval number YBU-2017C22.

### 2.2 Determination of fatty acid composition and content

Gas chromatography was performed using an Agilent GC7890A, with a flame ionization detector (FID) and a 100 m Supelco SP™-2560 fatty acid methyl ester (FAME) capillary column. The column box temperature was set to 140 °C for 5 min, increasing by 4 ${}^{\circ}C\;\min^{-1}$ to 240 °C , and held for 40 min. The detector and injection port temperatures were both set to 260 °C . Helium was used at 207 KPa; the injection volume was 1 µL , with a split ratio of 20:1 .

### 2.3 Paraffin sections

Tissues were fixed with 4 % formalin for 48 h at room temperature using a fixative volume that was 5–10 times the tissue volume. After trimming to appropriate sizes, tissues were placed in embedding cassettes. Deparaffinization was performed using two–three changes of xylene, each for 10 min. Sections were then hydrated in two changes of 100 % ethanol for 3 min each followed by 95 % and 80 % ethanol for 2 min each. They were then rinsed in distilled water. HE (hematoxylin and eosin) staining of paraffin sections was conducted, and the adipocytes' area and diameter were analyzed using the Image-Pro Plus 6.0 image analysis system (Hewitson et al., 2010)

### 2.4 RNA extraction and quantitative real-time polymerase chain reaction (PCR)

Total RNA from adipose tissue was extracted using an Eastep^®^ Super Total RNA Extraction Kit and the TRIzol reagent. RNA was adjusted to 500 ng for reverse transcription. Genomic DNA was removed by treating it with a gDNA Eraser, a potent DNA degradation agent, for 2 min at 42 °C followed by reverse transcription at 37 °C for 15 min and 85 °C for 5 s. Real-time polymerase chain reaction (PCR) involved an initial denaturation at 95 °C for 5 min followed by 40 cycles at 95 °C for 30 s, 60 °C for 60 s, and 72 °C for 60 s, then held at 4 °C . All analyses were repeated at least three times.

### 2.5 Primer design

Primers for genes such as *KDR*, *APOLD1*, *SCD1*, *SFRP4*, *FABP5*, *SCP2*, and *GAPDH* were designed according to the National Center for Biotechnology Information (NCBI) GenBank records using Oligo 6.0 and Primer 5.0. The primers, synthesized by Shanghai Bioengineering Co., Ltd., are listed in Table 1.

**Table 1:** Primer sequences used in quantitative PCR.

Gene symbol	Gene name	Primer (5' to 3')	Amplicon size (bp)
*KDR*	Kinase insert domain receptor	F: GCTTCTTGTCTCCAGACTGATCCT	151
		R: ATCACATCAGGACAGTAGGTAGGT	
*APOLD1*	Apolipoprotein L domain containing 1	F: GATGCAAGCATATCTATCCTGAAGG	144
		R: CAACTTCGCAGGACATCAGGCT	
*SCD1*	Stearoyl-CoA desaturase 1	F: GCCAACAACTCTGCCTTTATG	162
		R: CACCAATGACTGACCACCTG	
*SFRP4*	Secreted frizzled-related protein 4	F: GCGCTCACGGATGATGCTTCT	135
		R: CCTGCTGTTCGCTTCTTGTCCTG	
*FABP5*	Fatty-acid-binding protein 5	F: TGGCGCATTGGTTCAACATCAGG	188
		R: TGAACTGAGCTTGTTCATCCTCGC	
*SCP2*	Sterol carrier protein-2	F: TGAACTCCCTTTGCCTCCTTT	171
		R: CAGGTTCTATTCACCCAGCACTT	
*GAPDH*	Glyceraldehyde-3-phosphate dehydrogenase	F: CGTGTCTGTTGTGGATCTGACCTG	176
		R: CAACCTGGTCCTCAGTGTAGCCT	

### 2.6 Statistical analysis

All experiments in this study included six biological replicates. After the reaction, amplification and dissolution curves were drawn. Gene expression was quantitatively analyzed using the $2^{-\Delta \Delta \text{CT}}$ method. Data processing was performed using SPSS Statistics statistical software, with significant differences set to have P<0.05 .

## 3 Results

### 3.1 Fatty acid composition and content of adipose tissue in different parts of Yanbian yellow cattle

Fatty acid analysis was conducted on kidney, abdominal, omental, and subcutaneous adipose tissue of Yanbian yellow cattle, yielding intriguing findings, as detailed in Table 2. Abdominal gas chromatography revealed the presence of 16 distinct fatty acids, including arachidonic acid, caprylic acid, lauric acid, myristic acid, myristoleic acid, pentadecanoic acid, palmitic acid, palmitoleic acid, heptadecanoic acid, stearic acid, linolelaidic acid, elaidic acid, linoleic acid, γ -linoleic acid, behenic acid, and eicosatrienoic acid. Subcutaneous adipose tissue analysis unveiled the additional presence of henicosanoic acid and behenic acid (C22:0). Notably, caprylic acid (C8:0) was detected in both kidney and omental adipose tissue, bringing the total number of detected fatty acids to 19, surpassing the scope of previous studies which also delved into fatty acid profiles across cattle breeds (Pećina and Ivanković, 2021; Bartoň et al., 2011).

The concentration of palmitic acid (C16:0) in SFAs was observed to be higher across kidney, abdominal, omental, and subcutaneous adipose tissues, with values of 197.62, 194.49, 205.17, and 170.86 $\mathrm{mg}\;g^{-1}$ , respectively. Although, the differences were not statistically significant ( P>0.05 ). This finding aligns with those reported by Bartoň et al. (2021), and Kelly et al. (2014) in other cattle breeds, indicating a consistent pattern in fatty acid composition across breeds. Stearic acid (C18:0) was the second-most-abundant SFA, with concentrations of 261.29, 90.34, 244.28, and 69.76 $\mathrm{mg}\;g^{-1}$ , respectively. Among MUFAs, elaidic acid (C18:1C) showed higher levels in all tested tissues, with the highest concentration observed in abdominal adipose tissue (240.17 $\mathrm{mg}\;g^{-1}$ ). This is in contrast to previous studies reported about by Kelly et al. (2014), where other MUFAs were more predominant. In the PUFA category, γ -linolenic acid and arachidonic acid (C18:3n-6) showed similar distributions across the tissues but, again, without significant differences ( P>0.05 ).

**Table 2:** Composition and content of adipose tissue fatty acid in Yanbian yellow cattle ( $\mathrm{mg}\;g^{-1}$ ).

Fatty acid	Structure	Kidney fat	Abdominal fat	Omental fat	Subcutaneous fat
Caprylic acid	C8:0	0.06±0.005	0	0.05±0.005	0
Capric acid	C10:0	$0.44\pm 0.02^{a}$	$0.29\pm 0.01^{c}$	$0.37\pm 0.01^{b}$	$0.36\pm 0.01^{b}$
Lauric acid	C12:0	$0.51\pm 0.08^{a}$	$0.30\pm 0.03^{b}$	$0.40\pm 0.04^{\text{ab}}$	$0.37\pm 0.015^{\text{ab}}$
Myristic	C14:0	$26.51\pm 1.63^{a}$	$22.65\pm 0.06^{\text{ab}}$	$23.62\pm 1.85^{\text{ab}}$	$19.25\pm 0.12^{b}$
Myristoleic	C14:1	$1.09\pm 0.03^{c}$	$6.43\pm 0.23^{a}$	$1.12\pm 0.33^{\text{cd}}$	$4.75\pm 0.37^{b}$
Pentadecanoic	C15:0	5.67±1.74	2.25±0.03	5.47±1.90	3.17±0.41
Palmitic	C16:0	197.62±2.34	194.49±11.60	205.17±14.88	170.86±12.40
Palmitoleic	C16:1	$18.93\pm 1.56^{c}$	$34.16\pm 1.84^{b}$	$17.19\pm 0.56^{\text{cd}}$	$38.84\pm 3.4^{\text{ab}}$
Heptadecanoic	C17:0	13.03±2.87	5.96±0.14	12.97±2.81	6.73±1.19
Stearic	C18:0	$261.29\pm 2.08^{a}$	$90.34\pm 4.7^{c}$	244.28±1.27 a ${}^{b}$	$69.76\pm 7.13^{d}$
Linolelaidic	C18:1T	$24.87\pm 2.90^{a}$	$9.49\pm 0.24^{c}$	23.54±2.57 a ${}^{b}$	$11.20\pm 1.58^{\text{cd}}$
Elaidic	C18:1C	184.08±9.63	240.17±0.79	179.64±11.81	237.08±44.47
Linoleic	C18:2C	$17.03\pm 1.47^{a}$	$7.68\pm 0.15^{b}$	$12.90\pm 3.53^{\text{ab}}$	$10.11\pm 0.27^{\text{ab}}$
γ -Linoleic	C18:3N6	3.27±1.41	0.56±0.02	2.57±0.75	0.94±0.09
Henicosanoic	C21:0	0.76±0.59	0	0.61±0.26	0.33±0.07
Eicosatrienoic	C20:2	0.33±0.05	0.18±0.01	0.29±0.09	0.33±0.05
Behenic	C22:0	0.87±0.60	0	0.57±0.28	0.17±0.01
Eicosatrienoic	C20:3N6	0.30±0.12	0.19±0	0.27±0.11	0.19±0.01
Arachidonic	C20:4N6	0.19±0.08	0.25±0.02	0.19±0.06	0.21±0.01
SUM		760.95	617.64	736.37	580.1
SFA		506.76	316.28	493.51	271.03
MUFA		227.07	297.93	234.39	301.98
PUFA		3.76	1	3.03	1.34

SFA=C8:0+C10:0+C12:0+C14:0+C15:0+C16:0+C17:0+C18:0+C21:0+C22:, MUFA=C14:1+C16:1+C18:1T+C18:1C+C18:2C, and PUFA=C18:3N6+C20:3N6+C20:4N6. Note that different superscripted letters in the same rows indicate significant differences of <0.05 .

### 3.2 Morphological observation of adipocytes in different parts of Yanbian yellow cattle

**Figure 1:**
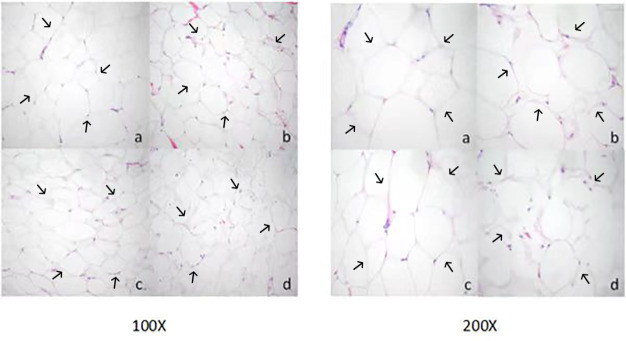
Morphological observation of adipose tissue cells in Yanbian yellow cattle. Paraffin sectioning and HE staining were used to prepare tissue sections of different parts, and the sections were observed at magnification of 100 and 200 times (a: kidney adipocyte, b: abdominal adipocyte, c: omental adipocyte, and d: subcutaneous adipocyte).

**Figure 2:**
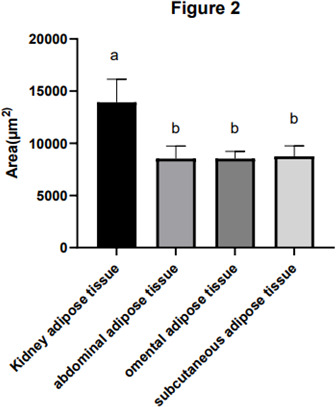
The area of the adipocytes of adipose tissue in Yanbian yellow cattle. The area of adipocytes in kidney, abdominal, omental, and subcutaneous adipose tissue was measured by the ImageJ processing system, and the statistical software used was GraphPad Prism 10. The x axis shows the types of adipose tissue detected, while the area of fat cells is shown by the y axis ( $µm^{2}$ ). In the figure, kidney adipose tissue, abdominal adipose tissue, omental adipose tissue, and subcutaneous adipose tissue are, respectively, shown from left to right, and the marks a, c, c, and b, respectively, indicate that the adipose cell area of kidney adipose tissue is significantly different from that of abdominal adipose tissue, omental adipose tissue, and subcutaneous adipose tissue. There was no significant difference in adipocyte area between abdominal adipose tissue and retinal adipose tissue, but there was a significant difference in adipose cell area between the subcutaneous adipose tissue and renal adipose tissue as well as between abdominal adipose tissue and retinal adipose tissue. Note that the difference between the same letters is not significant and that the difference between different letters is significant.

**Figure 3:**
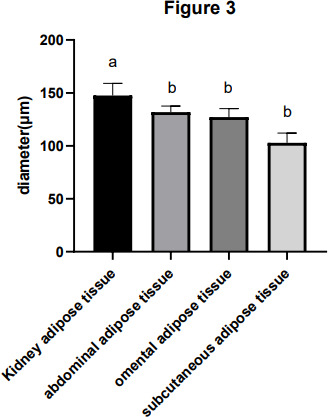
The diameter of the adipocytes of adipose tissue in Yanbian yellow cattle. The diameter of adipocytes in kidney, abdominal, omental, and subcutaneous adipose tissue was measured by ImageJ processing system and the statistical software used was GraphPad Prism 10. The x axis shows the types of adipose tissue detected, while the diameter of the adipocytes is shown by the y axis ( µm ). Note that the difference between the same letters is not significant and that the difference between different letters is significant.

Adipocyte morphology was assessed using hematoxylin and eosin (H&E) staining, with the area and diameter of adipocytes measured using the “ImageJ” image analysis system. The results, illustrated in Fig. 1, reveal larger adipocytes in kidney tissue, filled uniformly with large lipid droplets. In contrast, adipocytes in abdominal tissue were large, with irregular shapes and abundant lipid droplets. Omental adipose tissue displayed smaller, polygon-shaped adipocytes with decreased lipid droplets, while subcutaneous tissue featured adipocytes of uniform size and full cell walls, suggesting a tendency of larger cell synthesis. It is clear from Fig. 2 that the area occupied by the kidney adipose tissue was significantly larger than that of subcutaneous, abdominal, and omental adipose tissues ( p<0.05 ), whereas subcutaneous tissue showed the second largest area. Furthermore, the analysis presented in Fig. 3 shows a notable difference in the diameter of individual adipocytes within kidney tissue, representing a significant expansion compared to other tissues observed ( p<0.05 ). Our remarks on this distinguishing anatomical characteristic in Yanbian yellow cattle, characterized by enlarged adipocytes concealing substantial lipid droplets within kidney tissue, evidently deviate from findings documented in various other cattle breeds, as presented by Karolyi et al. (2009). These results underline the potential significance of such exclusive anatomical features as indicators of specific metabolic pathways or adaptive responses exclusive to Yanbian yellow cattle. It is a reasonable conclusion that these adaptations are subjective due to environmental factors or the breed's genetic heritage.

### 3.3 RNA extraction results

**Figure 4:**
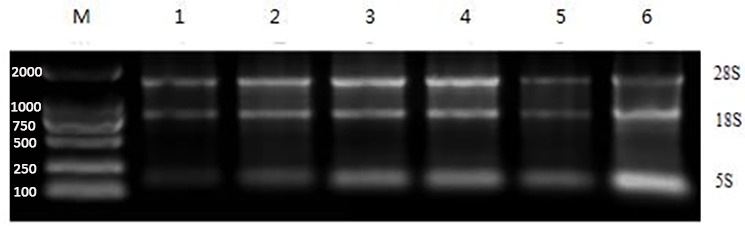
Gel photograph of total RNA of adipose tissues in different parts of Yanbian yellow cattle. Total RNA was extracted from different parts of adipose tissue with an Eastep^®^ Super Kit. 1: Posterior belly adipose tissue, 2: ribeye adipose tissue, 3: abdominal adipose tissue, 4: subcutaneous adipose tissue, 5: prothorax adipose tissue, 6: striploin adipose tissue, and M: DL2000 analytical quantity marker.

The Eastep^®^ Super Kit was used for the extraction of RNA from a range of adipose tissues, including ribeye, abdominal, subcutaneous, posterior belly, striploin, and prothorax, as shown in Fig. 4. Furthermore, the TRIzol method was used to extract total RNA from the visceral tissue of Yanbian yellow cattle. After the total RNA was extracted, the purity and concentration of the product were determined by an ultra-fine ultraviolet spectrophotometer, and the samples of A260/A280 in the range of 1.8–2.0 and concentration >30 $\mathrm{ng}\;µL^{-1}$ were used for further processing. After extraction, a complete assessment of RNA purity was performed using agarose gel electrophoresis. Remarkably, distinct bands corresponding to 28S and 18S were detected, with the strength of the 28S band being approximately 2 times higher than that of the 18S band. This distinctive pattern serves as a reliable indicator of RNA of superior quality, devoid of any protein or DNA contamination, thereby ensuring its suitability for subsequent experimental analyses.

### 3.4 Relative level of expression of crucial genes in different regions of adipose tissue

**Figure 5:**
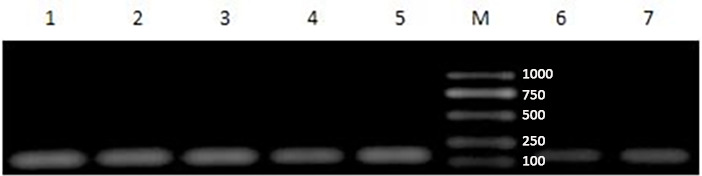
Adipose tissue PCR amplification electrophoresis results. 1: *KDR* (kinase insert domain receptor), 2: *APOLD1* (apolipoprotein L domain containing 1), 3: *SCD1* (stearoyl-CoA desaturase 1), 4: *SFRP4* (secreted frizzled-related protein 4), 5: *FABP5* (fatty-acid-binding protein 5), 6: *SCP2* (sterol carrier protein-2), 7: *GAPDH* (glyceraldehyde-3-phosphate dehydrogenase, used as the internal control), and M: DL2000 molecular marker.

**Figure 6:**
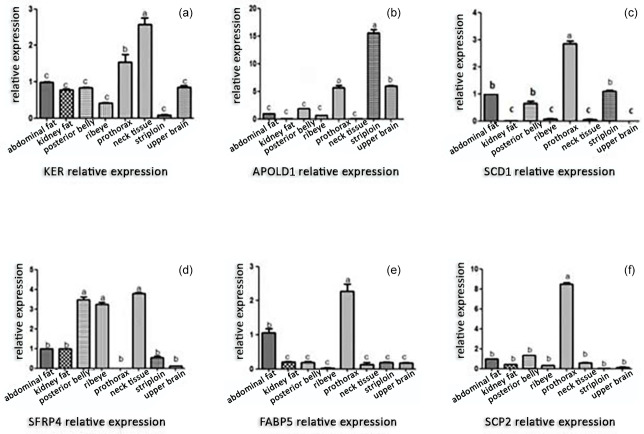
Gene expression in different adipose tissues of Yanbian yellow cattle. After a real-time PCR reaction, the adipose tissues in different parts are on the x axis and the relative expression is shown on the y axis. **(a)** The level of expression of the *KDR* gene in adipose tissues of prothorax and neck tissue is higher than that in kidney, abdominal, posterior belly, ribeye, striploin, and upper brain of adipose tissues ( p<0.05 ); **(b)** the level of expression of the *APOLD1* gene is the highest in striploin adipose tissues ( p<0.05 ); **(c)** the level of expression of the *SCD1* gene is the highest in prothorax adipose tissues ( p<0.05 ); and **(d)** the level of expression of the *SFRP4* gene in adipose tissues of the posterior belly, ribeye, and neck is higher than that of five other parts of adipose tissue ( p<0.05 ). **(e)** The *FABP5* gene has the highest expression in prothorax adipose tissue, followed by abdominal adipose tissue ( p<0.05 ) and **(f)** the level of expression of the *SCP2* gene is the highest in prothorax adipose tissues ( p<0.05 ). The panels show results for *KDR* (kinase insert domain receptor), *APOLD1* (apolipoprotein L domain containing), SCD1 (stearoyl-CoA desaturase 1), *SFRP4* (secreted frizzled-related protein 4), *FABP5* (fatty-acid-binding protein 5), and *SCP2* (sterol carrier protein-2), respectively. Note that the difference between bars labeled with the same letters is not significant and that the difference between those labeled with different letters is significant.

The inspection of gene expression level within adipose tissues discloses a fascinating distinction in their relative abundance. The adipose tissue expression of these genes is validated by the PCR amplification findings shown in Fig. 5. Evidently, there are discernible variations between various tissue types in the expression profiles of the *KDR*, *APOLD1*, *SCD1*, *SFRP4*, *FABP5*, and *SCP2* genes (Fig. 6). Comparing prothorax and neck tissues to other anatomical regions, the *KDR* gene was strongly expressed ( P<0.05 ), whereas the *APOLD1* gene was highly expressed in striploin tissue ( P<0.05 ). These findings are in line with earlier research by Ohsaki et al. (2009), who looked at how genotypes affected the content of fatty acids. Furthermore, it is important to carefully analyze the variations in the levels of gene expression for *APOLD1*, *SCD1*, *SFRP4*, *FABP5*, and *SCP2* in different adipose tissues. These genes are key in essential biological functions such as adipogenesis, lipid metabolism, and energy regulation. The considerable variation in their level of expression across different tissue types likely reflects the composite interplay of genetic, nutritional, and environmental factors that shape the characteristics of adipose tissue in Yanbian yellow cattle.

## 4 Discussion

Fatty acids play a crucial role in human health. Therefore, the composition and content of fatty acids in meat are very important. It is an important index in measuring nutritional value and whether meat is edible. SFAs, mainly C14:0, C16:0, and C18:0, are widely found in beef fat. The fatty acid content noted as the highest in this experiment includes C14:0, C16:0, C16:1, C17:0, C18:0, and C18:1, which are also the main fatty acids in beef from different production systems (Lan et al., 2014). Nearly half of the total bovine meat fatty acid content is mainly comprised of saturated fatty acids. A fast Gas chromatography-flame ionization detection (GC-FID) analysis, with a runtime of less than 7 min, quantified and identified these fatty acids from 25 heads of cattle. Based on their findings, the main fatty acid in all byproducts of different animals was oleic acid (C18:1 cis-9), with palmitic acid (C16:0) being the second major fatty acid followed by stearic acid (C18:0), palmitoleic acid (C16:1), myristic acid (C14:0), and linoleic acid (C18:2) (Marzocchi et al., 2018).

Some researchers examined the fatty acids in adipose tissue around the kidneys of Gannan black yak, finding higher contents of C16:0 and C18:1 and a slightly lower content of PUFA (Woollett et al., 1992). In this experiment, the polyunsaturated fatty acid content in kidney fat was 3.76 $\mathrm{mg}\;g^{-1}$ , which is inconsistent with the above results, possibly due to differences in sampling location and breed. This suggests potential differences in the regulatory mechanisms of intramuscular fat deposition between species. The capability to store intramuscular fat (IMF) is a characteristic that varies greatly in beef cattle. In breeds with a diminished inclination to accumulate IMF, meat quality may be compromised due to fat's contribution to organoleptic attributes, such as juiciness and flavor (Soret et al., 2016). Therefore, it is particularly important to discuss the content and composition of fatty acids in beef.

The dynamic changes in the physiological function of adipose tissue directly determine the quality and value of beef (Baik et al., 2023). It has been confirmed that the size and function of adipocytes are closely related to the distribution of adipose tissue and that the volume of adipocytes around organs is obviously larger than that of subcutaneous adipocytes. The results of this study showed that the kidney and subcutaneous adipocytes were large and uniform in size and that, at the same time, the diameter of individual adipocytes in kidney adipose tissue was also larger, corroborating previous findings. Different studies recommend using collagenase digestion to separate human abdominal subcutaneous adipose tissue cells,, omental adipose tissue cells and other tissue cells. It was found that abdominal subcutaneous adipose tissue cells are larger than omental adipocytes. There was no significant difference between the diameter of subcutaneous adipocytes and omental adipocytes, which is inconsistent with the above results; the reason may be the difference in species. Novel candidate genes for obesity are strongly related to preadipocyte number, body fat mass regulation, and adipocyte size in rats (Weingarten et al., 2016). Abdominal, visceral, and subcutaneous fat share a common metabolic feature, and their metabolites can be transported to the liver via the portal vein. Besides, the functions of abdominal adipose tissue and subcutaneous adipose tissue in receptor distribution, post-receptor signal transduction, and expression and activity of lipid metabolism-related genes are functionally different (Samaras et al., 2010).

With the deepening of molecular biology techniques, studying genes related to lipid metabolism can provide an important theoretical basis for regulating intramuscular fat. The current examination validates that *FABP5* is a critical regulator of MET- and E2-induced *SREBP-1c* gene expression that leads to milk fat synthesis (P. Li et al., 2018). Genetic and nutritional factors affect the expression of *FABP*s, and muscle tenderness and *FABP*s regulate marbling. It was also reported that *FABP5* may exert a pro-proliferative role in clear cell renal cell carcinoma. It can also be associated with malignant progression and tumor genesis in human beings (Lv et al., 2019).

*SCD1* is a rate-controlling enzyme that catalyzes the synthesis of MUFAs in the endoplasmic reticulum. It maintains the body's demand for MUFAs and plays a vital role in regulating the fatty acid composition of the body. The study showed that the *SCD1* gene is highly expressed in prothorax adipose tissue, abdominal adipose tissue, posterior belly adipose tissue, and striploin adipose tissue. The expression of *SCD1* reflects the synthesis of MUFAs in fat cells, and its activity determines the composition of lipid membrane phospholipids and triglycerides, *SCD* regulates membrane fluidity, alters cell membrane function, leading to changes in lipid metabolism and obesity (Scollan et al., 2006). Stearoyl-CoA desaturase (*SCD*) 1 catalyzes the rate-limiting reaction of monounsaturated fatty acid (MUFA) synthesis and plays an important role in the development of obesity. It was also found that the expression of the *SCD1* gene in muscle tissue of different breeds of cattle is different, and with the difference in MUFA and conjugated linoleic acid (CLA) content in muscle tissue, the expression of the *SCD1* gene and fatty acid desaturation index are further explored. There was a significant positive correlation, and the correlation between the *SCD5* gene expression and fatty acid desaturation index was poor.

Secreted frizzled-related protein 4 (*SFRP4*) is a member of the *SFRP* family that acts as soluble modulators of Wnt signaling pathway. Notably, *SFRP4* levels were found to exhibit a 2-fold increase in obese populations compared to non-obese counterparts, with abdominal subcutaneous adipose tissue evolving as a main contributor to the heightened circulating levels of *SFRP4* (Kumar Shrewastwa et al., 2023). In our investigation, we observed an elevated level of expression of *SFRP4* in adipose tissues localized within the posterior belly, ribeye, and neck. *SCP2*, recognized as a nonspecific lipid transporter, assumes a crucial role in facilitating intracellular lipid transport and metabolism and is often implicated in conditions such as lipid abnormalities (Li et al., 2016). Its functional flexibility expands to acting as a carrier for soluble sterols, accurately binding to these molecules. Similarly, it shows a widespread distribution throughout several cellular sections, including peroxisomes, mitochondria, endoplasmic reticulum, and cytosol (Dai et al., 2023; Gallegos et al., 2001). *APOLD1*, characterized by its compound functional domains, is generally distributed across several organs such as brain, kidney, pancreas, liver, and plasma. This widespread distribution point towards a potential secretory role of *APOLD1*, potentially involved in the secretion of signaling peptides. The intricate process of fat metabolism in animals incorporates a complex interaction between numerous genes. The complex fatty acid profile and dissimilar morphology of adipocytes witnessed in Yanbian yellow cattle, together with cautious analysis of gene expression, provide fruitful ground for future research into the relations between environmental and genetic causes that influence these traits. Understanding these dynamics is central for breeding programs envisioned to improve meat quality and nutritional value. Besides that, the information gained from our study can be used for the development of dietary interventions to promote human health given the vital role of fatty acid profiles in disease prevention and overall human health. Additionally, our study highlights the need for more research into the genetic foundation of these traits. The prominent expression of specific genes within particular adipose tissues suggests a genetic inclination that could be utilized in targeted breeding approaches. In addition, unraveling the environmental factors that have an influence on these traits could ease the adoption of more sustainable and effective practices in cattle farming.

## 5 Conclusion

In conclusion, Yanbian yellow cattle exhibit variations in both composition and content of fatty acids across different adipose tissue depots, including the kidney, abdominal, subcutaneous, and omental regions. Moreover, adipocytes display distinct morphological differences across these tissue types. Furthermore, the level of expression of *KDR*, *APOLD1*, *SCD1*, *SFRP4*, *FABP5*, and* SCP2* varies significantly among adipose tissues located in the kidney, abdominal, posterior belly, ribeye, prothorax, striploin, upper brain, and neck regions.

## Data Availability

The data presented in this study are available from the corresponding authors upon reasonable request.
